# *Corchorus olitorius* extract exhibit anti-hyperglycemic and anti-inflammatory properties in rodent models of obesity and diabetes mellitus

**DOI:** 10.3389/fnut.2023.1099880

**Published:** 2023-04-05

**Authors:** Kabelo Mokgalaboni, Wendy Nokhwezi Phoswa

**Affiliations:** Department of Life and Consumer Sciences, College of Agriculture and Environmental Sciences, University of South Africa, Florida, South Africa

**Keywords:** *Corchorus olitorius*, diabetes, obesity, anti-diabetic compounds, inflammation, dyslipidemia, antioxidant

## Abstract

Obesity and type 2 diabetes (T2D) are chronic conditions with detrimental impacts on the overall health of individuals. Presently, the use of pharmacological agents in obesity and T2D offers limited benefits and pose side effects. This warrant studies on remedies that are less toxic and inexpensive while effective in ameliorating secondary complications in obesity and T2D. Plant-based remedies have been explored increasingly due to their remarkable properties and safety profile. We searched for pre-clinical evidence published from inception until 2023 on PubMed, Scopus, Google, and Semantic scholar on *Corchorus olitorius* (*C. olitorius*) in both obesity and T2D. Our focus was to understand the beneficial impact of this plant-based remedy on basic glycemic, lipid, inflammatory, and biomarkers of oxidative stress. The evidence gathered in this review suggests that *C. olitorius* treatment may significantly reduce blood glucose, body weight, total cholesterol, triglycerides, and low-density lipoprotein (LDL) in concomitant with increasing high-density lipoprotein-cholesterol (HDL-c) in rodent models of obesity and T2D. Interestingly, this effect was consistent with the reduction of malonaldehyde, superoxide dismutase and catalases, tumor necrosis factor-alpha, interleukins, and leptin. Some of the mechanisms by which *C. olitorius* reduces blood glucose levels is through stimulation of insulin secretion, increasing β-cell proliferation, thus promoting insulin sensitivity; the process which is mediated by ascorbic acid present in this plant. *C. olitorius* anti-hyperlipidemia is attributable to the content of ferulic acid found in this plant, which inhibits 3-Hydroxy-3-methyl glutaryl coenzyme A (HMG-CoA) reductase inhibitors and thus results in reduced synthesis of cholesterol and increased hepatic LDL-c receptor expression, respectively. The present review provides extensive knowledge and further highlights the potential benefits of *C. olitorius* on basic metabolic parameters, lipid profile, inflammation, and oxidative stress in rodent models of obesity and T2D.

## 1. Introduction

**FIGURE 1:**
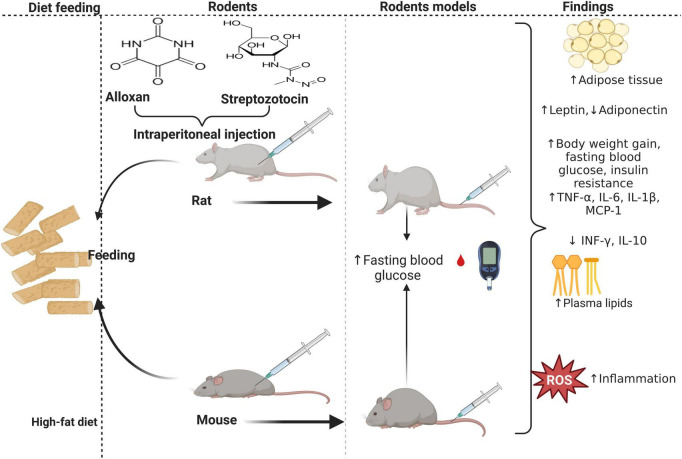
The effect of a high-fat diet (HFD) on inflammation and insulin resistance during obesity. HFD-fed mice develop while rats fed HFD concomitant to intraperitoneal injection of alloxan monohydrate or streptozotocin (STZ) develop diabetes. Rodents with diet-induced obesity or diabetes have enlarged adipocytes, elevating pro-inflammatory cytokines, adipokines, and lipid profiles, concomitant to decreased anti-inflammatory cytokines and adipokines.

Diabetes mellitus (DM), a chronic metabolic disorder, is characterized by an increased level of glucose in the blood. In an International Diabetes Federation (IDF) report of 2021, 537 million people were living with DM globally (1). Hyperglycemia observed in DM induces oxidative stress (2, 3) through stimulation of mitochondrial enzymes, resulting in the accumulation of reactive oxygen species (ROS) (4), which impair organ function (5). Furthermore, DM is also associated with high levels of triglycerides (TG), homocysteinemia, low-density lipoprotein-cholesterol (LDL-c), and reduced levels of high-density lipoprotein-cholesterol (HDL-c) (6, 7), which predispose type 2 diabetes (T2D) to the development of cardiovascular diseases (CVD) (6). In animal models of obesity or diabetes induced by a high-fat diet (HFD), a substantial increase in oxidative stress is present from the early stages of the disease (8, 9). This is accompanied by inflammation, impaired insulin secretion, action, and CVD development. In obesity, increased leptin and adipocytes are associated with an imbalance between pro and anti-inflammatory cytokines. Concomitant to that, resulting in inflammation, insulin resistance, and hyperglycemia (10) (Figure 1). Moreover, there is an impaired secretion of adipokines in obese states, and this increases the risk of CVD (11). There is an association between hyperglycemia, hyperglycemic-induced oxidative stress, inflammation, and the development and progression of T2D. Various reports have shown that chronic low-grade inflammation is associated with the risk of developing T2D and that sub-clinical inflammation contributes to insulin resistance and is linked to the characteristics of metabolic syndrome which include hyperglycemia. Oxidative stress induces the generation of inflammatory cytokines, and inflammation and further enhances the production of ROS (12, 13).

Notably, when exploring the efficacy of anti-diabetic drugs or therapies, paying attention to their antioxidant properties and effectiveness in improving the impaired lipid profile and glycemic control is crucial. The widely used T2D and dyslipidemic drugs such as Glucophage (metformin) (14) and statins (15) play a significant role in controlling these conditions; however, they are less tolerated in other patients due to their toxicity and severe adverse effects experienced by other patients (16, 17). Moreover, despite their availability, the rate of CVD complications associated with T2D is alarming globally, suggesting their limitations in cardiometabolic diseases (1, 18, 19). Hence, this warrants more investigation into alternative therapy and naturally occurring products that can regulate hyperglycemia and dyslipidemia with fewer side effects (20–23). Antioxidants are considered effective treatment due to their ability to neutralize free oxygen radicals (24, 25). Genistein, a polyphenolic isoflavone when used as monotherapy or in combination with metformin exhibits anti-inflammatory effects as demonstrated by decreased serum interleukin-6 (IL-6), tumor necrosis factor-α (TNF-α) and C-reactive protein (CRP) (26).

*Corchorus olitorius* L (jute mallow leaves) has been investigated extensively due to its antioxidant properties (27) in pre-clinical studies as an alternative treatment for T2D (28, 29). Previous evidence suggests that *C. olitorius* may exhibit protective effects against T2D, obesity, inflammation, and oxidative stress partly due to its chemical composition, such as vitamins A and ascorbic acid (AA) (30, 31), iron, calcium, and fibers (32, 33). Therefore, the *C. olitorius* antioxidant and anti-inflammatory properties may be a relevant target in treating different CVD and metabolic diseases. A study by Olusanya et al. (34) confirmed the anti-diabetic properties of *C. olitorius* in a rodent model of T2D. Interestingly, other studies have also shown a beneficial effect of *C. olitorius* on glucose and lipid level profiles in the same model animal of T2D (35, 36). Most recently, Lee and team (37, 38) demonstrated the anti-dyslipidemic properties of *C. olitorius* extract and reduced body weight in rodent models of obesity. However, while such benefits are widely reported, Aikawa (39) reported conflicting findings as *C. olitorius* aqueous solution showed no effect on FBG, insulin, and lipid profile in obese rodents. These contradicting findings show the limitation of *C. olitorius* as an agent to ameliorate hyperglycemia and dyslipidemia; therefore, this warrants more extensive research to understand the beneficial impact of this plant in obesity and T2D. Interestingly, *C. olitorius* extract given in a short period has proven safe and well-tolerated without side effects among T2D rodents (34). Although no clinical studies have been conducted to investigate the beneficial impacts of these plants on obesity and T2D, the present review aimed to review extensively and highlight the effects of *C. olitorius* in rodent models of obesity and T2D, focusing on basic metabolic parameters, glycemic control, lipid profile, inflammation, and oxidative stress.

## 2. Literature search, inclusion, exclusion criteria, and selection process

A comprehensive search for pre-clinical evidence published from inception to 2023 was done, and this was updated on the 11th March 2023 using PubMed, Scopus, Semantic, and Google Scholar using medical subheading (MesH) terms and text words such as “Jute leaves,” “*Corchorus olitorius,”* “molokhia,” “obesity,” and “diabetes.” There was no language restriction applied in the search in all databases. One hundred and thirteen records were identified from the databases. Pre-clinical studies were considered relevant if they evaluated the effect of *C. olitorius* on obesity or diabetes. Different doses and duration of intervention of *C. olitorius* in obese and diabetes rodent models were included. However, studies not on rodent models of obesity or diabetes were excluded. Before the screening process, 26 records were identified as duplicates by the Mendeley Reference Manager and were excluded. Following the initial screening of the title, abstract, and keyword, 30 records were excluded as the titles and abstract were irrelevant to the review. Out of the 57 records retrieved, eight were not on obese or diabetes rodent models, one was in human subjects, 13 were in phytochemical studies, seven were *in vitro* evidence, and 12 were reviews. Therefore, only 16 studies were found relevant; an additional four were retrieved by manually screening relevant studies. Therefore 20 relevant pre-clinical studies exploring the potential of *C. olitorius* extract as an anti-diabetic, anti-dyslipidemic, and anti-inflammatory agent in the rodent models of obesity and T2D were included in the review (Supplementary Figure 1 and Supplementary Tables 1A–F).

## 3. General overview of the included pre-clinical studies

From the evidence gathered, the studies were conducted in different countries; for instance, nine were conducted in Nigeria, 2 in India, Japan, and Korea, and one from Bangladesh, Egypt, Iraq, Northern Cyprus, and Côte d’Ivoire. The studies were conducted in different rodent strains, including Wistar rats, Albino rats, Sprague–Dawley rats, mice of C57BL/6N or J strain, Long Evans rats, and Otsuka Long-Evans Tokushima Fatty (OLETF) rats. Researchers induced these metabolic disorders through different methods, such as HFD and intraperitoneal injection of either streptozotocin (STZ) or alloxan monohydrate at different doses (Tables 1, 2). The special focus was centered on understanding how plants affect fasting blood glucose (FBG), body weight, and lipid metabolism and the mechanisms involved in inflammation and oxidative stress. T2D can lead to CVD (40); hence various rodent models have been used to understand the pathways and mechanisms implicated in obesity, T2D, and CVD. Most importantly, these experimental models are used to explore the properties of several pharmacological compounds against secondary complications associated with obesity and T2D (41).

**TABLE 1:** A summary of studies evaluating the impact of *Corchorus olitorius* in a rodent model of obesity.

References and country	Experimental model	Experimental outcomes
Lee et al. (37) Korea	High-fat diet (HFD)-induced obesity in C57BL/6N male mice orally treated with 2, 4 mg molokhia leaf polysaccharide fraction (MPF) (powder dissolved in water, at a ratio of 1:20 w/v) for 8 weeks	MPF4 (4 mg) significantly decreased body weight, adipocyte size, serum triglyceride (TG), low-density lipoprotein cholesterol (LDL-c) levels, and inflammatory cytokines.
Lee et al. (38) Korea	HFD-induced obesity in C57BL/6J male mice treated orally with 50, 100 mg (100 g powder dissolved in 2 liters of distilled water) water-soluble extract of *C. olitorius* for 8 weeks.	*C. olitorius s*ignificantly reduced hepatic lipid and body weight and inhibited colonic inflammation.
Aikawa et al. (39) Japan	Otsuka Long-Evans Tokushima Fatty (OLETF) rats with obesity and hyperphagia were orally treated with 3% *C. olitorius* aqueous solution (powder dissolved in 60% ethanol) for 20 weeks.	*C. olitorius* showed no significant difference in fasting blood glucose (FBG), insulin, and lipid profiles.
Airaodion et al. (35) Nigeria	Obese male albino rats orally treated with 3 ml methanolic extract *C. olitorius* (25 g powder dissolved into 250 ml of methanol) 12 hourly for 14 days.	*C. olitorius* significantly decreased FBG, total cholesterol (TC), LDL-c, and TG without significantly increasing high-density lipoprotein cholesterol (HDL-c).
Adon et al. (47) Côte d’Ivoire	Female obese Wistar rats were treated orally with 300 mg/kg of the aqueous extract from the roots of *C. olitorius* (30 g powder into 100 ml distilled water) twice a day for 18 days.	*C. olitorius* significantly decreased TG and increased the HDL-c level without significant weight differences.
Gomaa et al. (48) Egypt	HFD-induced obesity in male Sprague–Dawley albino rats, orally treated with 300, 400 mg/kg ethanolic extract of *C. olitorius* (1 g powder dissolved in 70% ethanol) for 8 weeks.	*C. olitorius* significantly decreased FBG, insulin, leptin, TC, TG, LDL-c, free fatty acid, interleukin (IL)-1β, and tumor necrosis factor-a (TNF-α) levels. Additionally, HDL-c and adiponectin levels significantly increased.
Wang et al. (28) Japan	HFD-induced obesity in C57BL/6 mice (null LDL) treated with 1 and 3% molokhia leaf powder (MLP), *C. olitorius* (powder dissolved in 60% ethanol aqueous solution) for 8 weeks.	*C. olitorius* significantly decreased TG, liver, and body weight.

T2D, type 2 diabetes; *C. olitorius, Corchorus olitorius*; HFD, high-fat diet; MLP, molokhia leaf powder; LDL, c-low-density lipoprotein cholesterol; HDL, c-high-density lipoprotein cholesterol; TNF, α-tumor necrosis factor-alpha; IL, 1β-interleukin-1-beta; MPF, molokhia leaf polysaccharide fraction; TG, triglyceride; TC-total cholesterol.

**TABLE 2:** A summary of studies evaluating the impact of *Corchorus olitorius* in rodent models of diabetes.

References and country	Experimental model	Experimental outcomes
Swayeh and Kadhim (49) Iraq	Streptozotocin (STZ)-induced diabetes rats fed a high-fat diet (HFD) and treated with 400 mg methanolic extract of *C. olitorius* (1,150 g powder dissolved in 60% ethanol) for 28 days.	*C. olitorius* significantly decreased fasting blood glucose (FBG), glycated hemoglobin (HbA1c), homeostasis model assessment of insulin resistance (HOMA-IR), total cholesterol (TC), triglycerides (TG), low-density lipoprotein cholesterol (LDL-c), and increased adiponectin and high-density lipoprotein-cholesterol (HDL-c).
Anyasor et al. (50) Nigeria	Alloxan-induced diabetes in male Albino rats received 80 g ethanolic extract of *C. olitorius* (100 g of the powder dissolved in 600 ml of 50% ethanol) for 14 days.	*C. olitorius* significantly decreased FBG, LDL-c, TC, and TG levels.
Ali et al. (51) Bangladesh	STZ-induced diabetes in Long Evans rats treated with 10 ml/kg ethanolic extract of *C. olitorius* (powder was dissolved in 96% ethanol) for 28 days.	*C. olitorius* significantly decreased serum FBG levels.
Mercan et al. (52) Northern Cyprus	STZ-induced diabetes in male Wistar rats orally treated with 250 ml/kg ethanolic extract of *C. olitorius* (powder dissolved in 96% ethanol) for 3 weeks.	*C. olitorius* significantly decreased FBG.
Mohammed et al. (53) Nigeria	Alloxan-induced diabetes in male albino rats orally treated with 400 mg/kg aqueous extract of *C. olitorius* (powder dissolved in distilled water) for 28 days.	*C. olitorius* significantly decreased FBG, TG, TC, and LDL-c, concomitant to an increased HDL-c.
Patil and Jain (54) India	STZ-induced diabetes in Wistar rats and orally treated with 100 and 200 mg/kg methanolic extract of *C. olitorius* (powder dissolved in methanol) for 21 days.	*C. olitorius* significantly decreased FBG and cholesterol levels.
Saliu et al. (55) Nigeria	STZ-induced diabetes in Wistar rats fed HFD orally treated with 100 mg/kg of jute leaf for 30 days.	*C. olitorius* significantly increased hepatic δ-ALAD, catalase and superoxide dismutase (SOD) activity, and decreased malonaldehyde (MDA).
Olusanya et al. (34) Nigeria	Alloxan-induced diabetes in Albino rats and treated orally with 200, 400, and 800 mg/kg ethanolic extract of *C. olitorius* (40 g powder dissolved in 2,500 ml of 80% ethanol) for 14 days.	*C. olitorius* significantly decreased FBG, TC, TG, and LDL-c and increased HDL-c.
Onyechi et al. (56) Nigeria	Alloxan-induced diabetes in male Albino rats treated orally with 100 and 300 mg/kg aqueous extract of *C. olitorius* (200 g of powder dissolved in 400 ml of distilled water) for 14 days.	*C. olitorius* significantly decreased FBG, body weight, TC, TG, and LDL-c, concomitant to increased serum HDL-c levels.
Omeje et al. (57) Nigeria	STZ-induced diabetes rats were treated orally with 50, 100, 150, and 200 mg/kg ethanolic extract of *C. olitorius* (450 g of powder dissolved in 1 liter of 70% aqueous ethanol) for 30 days.	*C. olitorius* at 150 mg/kg significantly decreased FBG, catalytic, and SOD activities.
Saliu et al. (58) Nigeria	STZ-induced diabetes in male Wistar rats fed HFD and treated with 10% of jute leaves for 30 days.	*C. olitorius* significantly decreased FBG, α-amylase, α-glucosidase, lipid peroxidation, TC, TG, and MDA levels.
Egua et al. (59) Nigeria	Alloxan-induced diabetes in male Albino rats orally treated with 125, 250, 500, and 1,000 mg/kg ethanolic extract of *C. olitorius* for 14 days.	*C. olitorius* significantly decreased FBG and HbA1c, coupled with increased insulin levels.

T2D, type 2 diabetes; TC, total cholesterol; TG, triglyceride; HFD, high-fat diet; LDL-c, low-density lipoprotein cholesterol; HDL-c, high-density lipoprotein cholesterol; SOD, superoxide dismutase; AST, aspartate aminotransferase; ALAD, aminolevulinic acid dehydratase; MDA, malondialdehyde; STZ, streptozotocin; FBG, fasting blood glucose; HOMA-IR, homeostasis model assessment of insulin resistance; HbA1c, glycated hemoglobin.

## 4. An overview of *C. olitorius*, components, bioavailability, and functional properties

*Corchorus olitorius* is a naturally occurring plant used as a vegetable in different African and Asian communities and belongs to the family of Malvaceae. For example, at least 97.7% of individuals in Uganda use *C. olitorius* for consumption (42). However, it is cultivated in other parts, including Australia, South America, and Europe, to be used in food products as additives and industrial applications. The nutritious quality and value of *C. olitorius* are attributable to its high level of vitamins, minerals, and phenolics (43). All components of the *C. olitorius* plant are useful; for example, dried leaves are used as herbal tea, green and fresh leaves are used as vegetables, and seeds are used as a flavoring agent.

**FIGURE 2:**
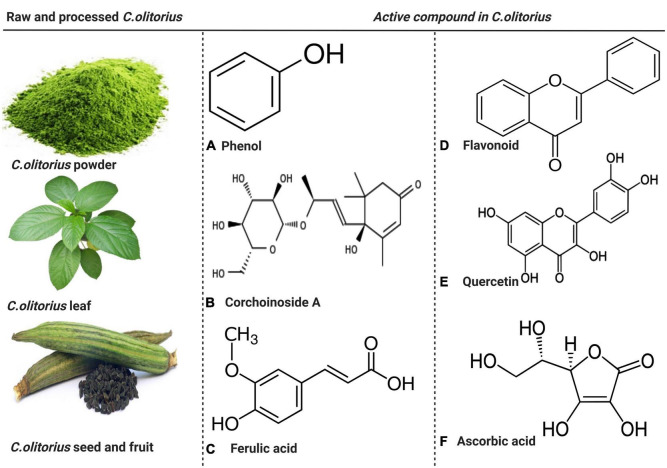
The raw and processed parts of *Corchorus olitorius* and active compound present in *C. olitorius*. Both raw and processed *Corchorus olitorius* contains active compounds such as phenol, corchoinoside A, ferulic acid, flavonoids, quercetin, and ascorbic acid.

Bioavailability refers to the amount of substance absorbed and reaching the circulation for body use (44). However, knowledge of the bioavailability of *C. olitorius* and its derivatives is limited. Considering the abundance of active compounds it contains, it is likely that its absorption mechanism would not differ from that of the active chemical compound it contains. This plant is rich in carbohydrates, fats, protein, iron, calcium, potassium, sodium, phosphorus, beta-carotene, thiamine, riboflavin, niacin, AA, triterpenes, sterols, fatty acid, phenolics, ionones, oxidase, chlorogenic acid, glycosides, saponins, tannins, and flavones (45). Its high content of various compounds (Figure 2) makes it a unique leafy vegetable with anti-diabetic, cardioprotective, and anti-inflammatory (46).

## 5. Effect of *C. olitorius* on the body weight and glucose metabolism in rodent models of T2D

Diabetes is characterized by hyperglycemia which is often coupled with body weight gain. This is observed in humans and the animal model impaired glucose tolerance (3, 60, 61). It is public knowledge that correct regulation of blood glucose levels and effective control of other primary metabolic indices such as body weight and insulin levels are crucial when controlling and managing T2D. Experimental evidence supports this antioxidant, *C. olitorius, in* regulating blood glucose levels (Table 2). For example, Anyasor et al. (50) demonstrated that using 80 g ethanolic extract of *C. olitorius* leaf for 13 days in T2D rats could improve glucose metabolism, as revealed by a significant decrease in plasma glucose in the treated group (99.0 mg/dL) compared to 313.5 mg/dL, *p* < 0.05 in the control group. Similarly, Mercan et al. (52) reported that the administration of 96% ethanolic extract of *C. olitorius* (250 mg/kg) for 3 weeks in STZ-induced T2D Wistar albino rats could significantly decrease FBG (297.83 mg/dL) when compared to untreated STZ rats (419.00 mg/dL, *p* < 0.05). The same study revealed a 0.44-fold decrease in FBG when baseline and post-treatment results were compared. Additionally, in Albino rats, treatment with 3 ml of methanolic extract of *C. olitorius* decreases FBG (35). Interestingly, FBG was significantly reduced when methanolic extract of *C. olitorius* was administered at 100 mg/kg (118.00 mg/dL), *p* < 0.005, or 200 mg/kg (120.00 mg/dL), *p* < 0.05 compared to control group 397.00 mg/dL (54). The effect was more predominant when a lower dose (100 mg/kg) of the methanolic extract was administered. Similar trends were observed in T2D Albino rats, as FBG was significantly reduced to 9.43 mg/dL at 200 mg/kg, 7.88 mg/dL at 400 mg/kg, and 6.05 mg/dL at 800 mg/kg of *C. olitorius* extract compared to the diabetic group without treatment (20.41 mg/kg), *p* < 0.05 (34). Similarly, Mohammed et al. (53) revealed a significant effect of 400 mg/kg of *C. olitorius* extract on reducing FBG (5.20 mg/dL) compared to diabetic control (10.30 mg/dL), *p* < 0.05. Another study that induced T2D through STZ in Wistar rats and administered 10% of *C. olitorius* also revealed a significant reduction in FBG levels (*p* < 0.05) (58). Consistently, Omeje et al. (57) also revealed that oral administration of ethanol extract of *C. olitorius* in rats at low or high doses (50, 100, 150, and 200 mg/kg) for 30 days still decreases FBG compared to the untreated group (138.1 mg/dL). However, a pronounced decrease was observed using 200 mg/dL of ethanol extract (96.5 mg/dL), which was deemed a high dose according to the study. Of interest was one study that demonstrated a significant decrease in various parameters associated with glycemic control. This study showed a reduction in FBG (271.50 mg/dL, *p* < 0.001), glycated hemoglobin (HbA1c) (4.23%, *p* < 0.001) when 400 mg/kg methanolic extract of *C. olitorius* compared to untreated T2D rats whose FBG and HbA1c were 408.90 mg/dL and 4.98%, respectively (49). A similar trend was observed by Egua et al. (59) as they demonstrated a significant decrease in FBG and HbA1c concomitant to an increased insulin level in Alloxan-induced T2D. This study used different doses, including 100, 250, 500, and 1,000 mg/kg, and all extracts showed a significant decrease (*p* < 0.05) in FBG. However, at 100 mg/dL, there was a pronounced decrease of 157.2 mg/dL compared to the control 183.6 mg/dL, *p* < 0.05.

**FIGURE 3:**
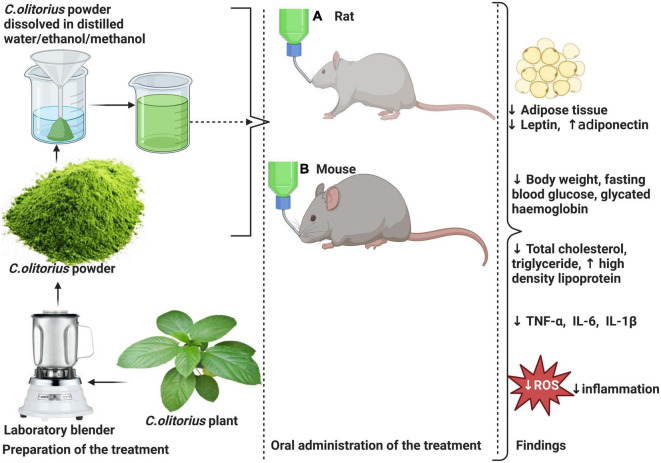
Effects of *Corchorus olitorius* in a rodent model of diabetes induced by HFD and intraperitoneal injection of streptozotocin (STZ) or monohydrate alloxan in rodents. This is followed by a moderate decrease in the size of adipose tissue and a subsequent decrease in leptin concomitant to increased adiponectin levels. *C. olitorius* reduce inflammation through the reduction of pro-inflammatory markers such as tumor necrosis factor-alpha (TNF-α), interleukin-6 (IL-6), and interleukin-1-beta (IL-1β). The anti-hyperglycemic potential of *C. olitorius* is demonstrated by a decrease in total cholesterol levels, triglyceride, low-density lipoprotein-cholesterol (LDL-c), and increased high-density lipoprotein (HDL-c). Also, reduced blood glucose and glycated hemoglobin demonstrate its effect on basic glycemic parameters.

According to the results by Onyechi et al. (56), *C. olitorius* administration in T2D Wistar significantly reduced FBG post-treatment (*p* < 0.05), and this was more predominant when 300 mg/kg instead of 100 mg/kg was administered. Moreover, the same study revealed a decrease in body weight in 100 and 300 mg/kg of *C. olitorius* extract 143.12 and 155.98 g, respectively, compared to the control group 245.65 g (Figure 3).

**FIGURE 4:**
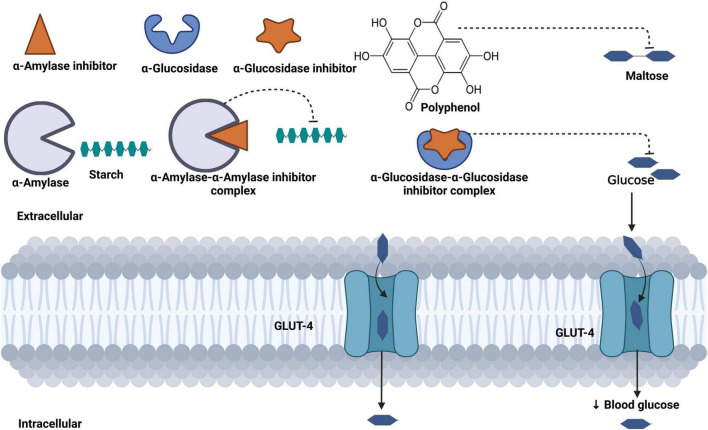
Mechanism of blood glucose regulation mediated by phenol content in *Corchorus olitorius*. Polyphenols inhibit starch-digesting enzymes (α-amylase, β-glucosidase), reduce the breakdown of starch into maltose, and further reduce the transportation of glucose in the bloodstream.

The reduced hyperglycemia in these rodent models may be attributed to the phytochemical content of *C. olitorius*, such as flavonoids (62–65), saponins (66), and thiamine (67), which are proven to have hypoglycemic properties. Moreover, *C. olitorius* is rich in polyphenols, which have inhibitory effects on α-amylase and glucosidase, enzymes essential for glucose catabolism (30, 55). Slow intake, digestion, and absorption of a starch-containing diet remain important mechanisms for reducing blood glucose. Therefore, inhibition of pancreatic α-amylase and glucosidase reduces the breakdown of carbohydrates into glucose and absorption of glucose in the small intestine, thereby reducing hyperglycemia (Figure 4) (68, 69).

## 6. Effect of *C. olitorius* on the body weight, adipocytes, leptin, adiponectin, and glucose metabolism in a rodent model of obesity

According to the Centers for Disease Control and Prevention (CDC), obesity is defined by increased body mass index (30 kg/m^2^) (70). It is characterized by an excessive accumulation or hypertrophy of adipocytes, partly due to increased calorie intake, without physical activities. An overview of evidence showing the effects of *C. olitorius* on basic glycemic control is presented in Table 2. According to a study by Lee et al. (38), the use of HFD to induce obesity in C57BL/6J mice and subsequent administration of 100 mg *C. olitorius* extract showed a significant decrease in body weight. The same group revealed that administration of *C. olitorius* treatment at 4 mg/kg molokhia leaf polysaccharide fraction for 8 weeks reduces body weight in C57BL/6N mice fed HFD (37). However, this time around, the effects were accompanied by a significant decrease in the size of adipocytes. This reflects the potential effect of this antioxidant plant during obesity.

Consistently, adipocyte in obesity is associated with the recruitment of monocytes and inflammation, which are central to the development of insulin resistance and further CVD (71). Therefore, a reduced adipocyte size following *C. olitorius* administration could minimize the risk of insulin resistance in obesity and alleviate secondary complications. Additionally, in Sprague–Dawley rats fed HFD to induce obesity, the administration of *C. olitorius* at either a dose of 300 mg/kg or 400 mg/kg showed a significant decrease in serum glucose and insulin (48). The same study also showed a significant reduction in leptin concomitant to an increase in adiponectin; this is partly because of the direct relationship between adipocytes in obesity and its influence on leptin levels. The high content of phytol found in *C. olitorius* reduces leptin, which is associated with the reversal of insulin resistance in obesity. This action is mediated by the inhibition of inflammatory cytokines that trigger insulin resistance. According to a study by Yagishita et al. (72), hypothalamic-related oxidative stress induces leptin resistance, which results in insulin resistance and obesity.

Conversely, adipocyte macrophages contribute to increased levels of inflammatory cytokines such as TNF-α, IL-1β, and IL-6. These inflammatory cytokines induce insulin resistance by activating the suppressors of cytokine signaling proteins in insulin-target tissues (73). Both leptin and adiponectin are important in obesity through indirect regulation of food intake and body weight (74). In fact, leptin reduces body weight by decreasing food intake. However, obesity is associated with an elevated level of leptin, which can stimulate hunger, increase food intake and impair metabolism; this exacerbates obesity the latter results in extra body weight gain in the form of adipose storage (75).

On the other hand, adiponectin, an adipokine produced by adipose, exhibits antioxidant and anti-inflammatory properties that regulate blood glucose, lipid metabolism, and insulin sensitivity (76). Notably, alteration in the adiponectin gene expression and its receptors in obesity is associated with decreased levels and sensitivity; the latter results in insulin resistance, exacerbating hyperinsulinemia. In C57BL/6 with knocked low-density lipoprotein, receptor (LDLR-/-) mice fed HFD to induce obesity when given 1 and 3% *C. olitorius* for 8 weeks showed a significant decrease in body 26.7 and 27.2 g, respectively, when compared to control 28.3 g, *p* = 0.032 (28). In addition, the same study revealed a significant decrease in liver weight at both doses, 1.5 and 1.43 g, compared to the control, 1.74 g, *p* = 0.001. Obese status is associated with fat accumulation in the liver, resulting in liver hypertrophy, chronic inflammation, and cirrhosis.

In a recent study by Airadion et al. (35), 3 ml oral administration of *C. olitorius* in obese Albino rats; significantly decreased FBG when compared to the control (*p* < 0.05). Similarly, the intergroup difference also revealed a significant decrease in FBG post-treatment compared to pre-treatment (*p* < 0.05). It is assumed that the potential effect of *C. olitorius* in ameliorating hyperglycemia may be mediated by AA found in *C. olitorius*, as it improves insulin sensitivity (77). AA is an antioxidant, and a previous meta-analysis has shown that AA effectively reduces blood glucose (78).

Despite other studies showing the beneficial effects of *C. olitorius* on body weight, FBG, and insulin sensitivity, conflicting results were reported by Aikawa et al. (39), who found that administration of a 3% *C. olitorius* in obese rats had no significant effect on FBG and insulin. However, it is important to note that this study used an obese-hyperphagia model of OLETF rats (39). Therefore, their findings may be influenced by the hyperphagia stage as it is associated with increased ghrelin hormone in obesity (79, 80). Ghrelin is known to increase appetite (81), and therefore, it is likely to exacerbate obesity and insulin resistance despite the administration of *C. olitorius.*

Similarly, another study found contrasting findings as it reported no evidential improvement in hyperglycemia and body weight following the administration of *C. olitorius*. For example, 18 days of 300 mg/kg of *C. olitorius* in Wistar rats showed moderate body weight gain (+ 0.11 g) compared to control (+ 0.48 g) despite showing consecutive weight loss at days 6, 9, and 12. However, this was not statistically significant (*p* > 0.05) (47). Although these studies did not show a significant difference *per se*, the results may be explained partly by the gender and the strain and number of the rats used; for instance, other studies reported that they used males, while this researcher used female rats. A study by Taraschenko et al. (82) reported that female rats, when fed HFD, do not become obese and do not respond to treatment compared to male rats. Additionally, the small sample size (*n* = 3 rats per group) might have influenced the statistical power of the investigation. It has been shown previously that a small sample size affects the power and effect size of experiments in pre-clinical and clinical studies (83).

## 7. Effect of *C. olitorius* on lipid profile in rodent models of T2D

The lipid profile encompasses TC, TG, LDL-c, and HDL-c. These remain the primary markers of dyslipidemia in obesity and diabetes. The evidence gathered in this review suggests the potential of *C. olitorius* as anti-hyperlipidemia. The general overview of the studies reporting on the impact of *C. olitorius* in alleviating hepatic lipid accumulation to improve dyslipidemia in experimental models of T2D is presented in Table 2. An impaired lipid profile in T2D is associated with dyslipidemia, atherosclerosis, and an increased risk of CVD (61). According to Anyasor et al. (50), 80 g of *C. olitorius* in T2D Albino rats improved triglyceride (82.44 mg/dl), TC (108.6 mg/dl), and LDL-c (55.98 mg/dL) when compared to control TG (127.03 mg/dl), TC (137.51 mg/dl), LDL-c (76.82 mg/dl), *p* < 0.05. Similarly, when Wistar rats were given about 100–200 mg/kg of methanolic extract of *C. olitorius* for 21 days, they showed a decrease in TC level (54). However, only 200 mg/kg of the extract demonstrated a significant decrease in TC (125.0 mg/kg) compared to the control 210.0 mg/kg, *p* < 0.05. A 14-day administration of either 200, 400, or 800 mg/kg of *C. olitorius* in T2D Albino rats also showed a significant decrease (*p* < 0.05) in TC, TG, LDL-c, total protein, concomitant to an increased HDL-c (34). Consistently, treatment with *C. olitorius* in HFD-fed STZ-induced diabetic rats, TC and TG levels significantly decreased when compared to an untreated group (*p* < 0.05) (58). The findings are corroborated by Onyechi et al. (56) who reported a decrease in serum TC, TG, and LDL-c, concomitant to increased HDL-c levels (*p* < 0.05). A similar trend was observed by Swayer and Kadhim (49), who also demonstrated a significant decrease in TC (73.05 mg/dL), TG (85.4 mg/dL), LDL-c (36.07 mg/dL), and coupled with increased HDL-c (19.9 mg/dL) (*p* < 0.05).

According to the evidence synthesized in this review, we are of the opinion that *C. olitorius* has the potential for use as an alternative treatment to curb hyperlipidemia in T2D (Figure 4). While this evidence is obtained from pre-clinical studies, it would be better to understand the mode of action of this plant in ameliorating hyperlipidemia. While such benefits are acknowledged in ameliorating dyslipidemia, it seems they are attributable to ferulic acid found in this plant, partly because it can inhibit 3-Hydroxy-3-methyl glutaryl coenzyme A (HMG-CoA) reductase inhibitors and thus resulting in reduced synthesis of cholesterol and hence low levels of TC and LDL-c. Moreover, the antioxidant effect of ferulic acid may also decrease lipid peroxidation, causing decreases in the oxidized LDL-c (84).

## 8. Effect of *C. olitorius* on lipid profile in rodent models of obesity

The general summary showing the potential of *C. olitorius* as anti-hyperlipidemia is presented in Table 2. For example, a study by Gomaa et al. (48) demonstrated that *C. olitorius* at 300 and 400 mg/kg doses for 8 weeks significantly reduced plasma TG levels in 8.27 and 6.28 mg/dL, respectively, compared to untreated HFD-fed Sprague–Dawley obese rats 152.25 mg/dL, *p* < 0.05. Another study also corroborates the prior findings as reported that *C. olitorius* supplementation at 300 mg/kg for 18 days could reduce TG (0.56 mg/dL) levels concomitant to an increase in the HDL-c (0.26 mg/dL) level compared to control (0.8 and 0.39 mg/dL), *p* < 0.05, respectively (47). In C57BL/6N, male mice on 8-week HFD for induction of obesity showed that 4 mg/kg of *C olitorius* is effective as it significantly reduced serum TG and LDL-c levels (*p* < 0.05) (37). C57BL/6 (LDLR-/-) mice fed HFD for 8 weeks and treated with 3% of *C. olitorius* revealed significantly decreased TG levels 192 mg/dL compared to control (262 mg/dL), *p* = 0.009 (28). Albino rats administered with 3 ml of *C. olitorius* also demonstrated a beneficial impact by reducing TG, TC, and LDL-c without significant changes in HDL-c levels, *p* < 0.05 (35).

Contradicting results were reported by Lee et al. (38) who revealed no significant difference in TG and TC (*p* > 0.05) following treatment with 100 mg of *C. olitorius* in HFD-fed C57BL/6J mice. Similarly, another study showed limited potential effects of *C. olitorius* extract and showed no significant difference in serum and liver TG, TC (*p* > 0.05) in the obese-hyperphagia model of OLETF rats (39). Nevertheless, experimental evidence summarized in this review suggests that *C. olitorius* may improve lipid metabolism. It seems *C. olitorius* can ameliorate hyperlipidemia through various mechanisms; for example, the beneficial effects on lipid profile could be attributed to *C. olitorius* phenolic content, with antioxidative properties, which include stimulation of lipid metabolism in the liver, removal of excess fat in adipocytes and the liver, and lower plasma and hepatic lipid levels, resulting in an anti-obesity effect (28). In addition, the saponins content in the root of *C. olitorius* may contribute to its anti-hyperlipidemic and anti-hypercholesterolemia (35, 85). Furthermore, evidence suggests that *C. olitorius*’s ability to reduce the TC and TG levels in the body is attributable to its alkaloid content, which increases the hepatic LDL-c receptor expression and inhibits lipid synthesis in the liver (86–88).

## 9. The general overview of *C. olitorius* on oxidative stress and inflammation in rodent models of obesity and T2D

Chronic inflammation induced by obesity can result in β-cell dysfunction, insulin resistance, and T2D (89). Primary pro-inflammatory markers such as TNF-α, IL-6, and IL-8 induce inflammation and are central to the pathogenesis of CVD associated with obesity and T2D. In a homeostatic state, there is a general balance between the ROS produced and the ability of the body to neutralize these active molecules. However, excessive ROS generation can damage the structure and triggers an inflammatory response and oxidative stress that initiates the development of T2D (90) and cardiovascular disease (91). Important widely used biomarkers of oxidative stress include malonaldehyde (MDA), superoxide dismutase (SOD) and enzyme δ-aminolevulinic acid dehydratase (ALAD) were reported in the gathered evidence. Antioxidants are known to counteract the reactive response generated by ROS; this ameliorates oxidative stress by scavenging free radical molecules (92). A report by Saliu and the team showed a significant decrease in MDA (*p* < 0.05) following treatment with 100 mg/kg *C. olitorius* extract in T2D (55). However, the same study revealed a significant increase in catalase, SOD, and ALAD (*p* < 0. 05) compared to the untreated T2D group. Moreover, another study reported a significant decrease (*p* < 0.05) in MDA when 10% jute leave was administered in T2D rats (58).

Similar observations were reported by another group of researchers who showed decreased SOD activities in T2D rats treated with 150 mg/kg ethanolic extract of *C. olitorius* (118.3 IU/L) compared to the untreated group (216.1 IU/L). Interestingly when the baseline was compared to post-treatment, the same study at 150 mg/kg registered a 0.5-fold decrease (230.8 IU/L) in SOD activity (57). The same study reported a decrease in catalase activity in the treated group (97.8 IU/L) compared to the untreated (100.6 IU/L). The activities of these enzymes are crucial in protecting cell integrity from oxidative stress. Therefore, the evidence gathered in this review suggests that *C. olitorius* may also reduce oxidative stress in T2D individuals. It has been confirmed in rodent models that *C. olitorius* significantly ameliorates oxidative stress (29, 55, 93). The antioxidant property of *C. olitorius* is attributable to flavonoids, AA (31, 94), carotenoids (45), and phenols contents (95).

**FIGURE 5:**
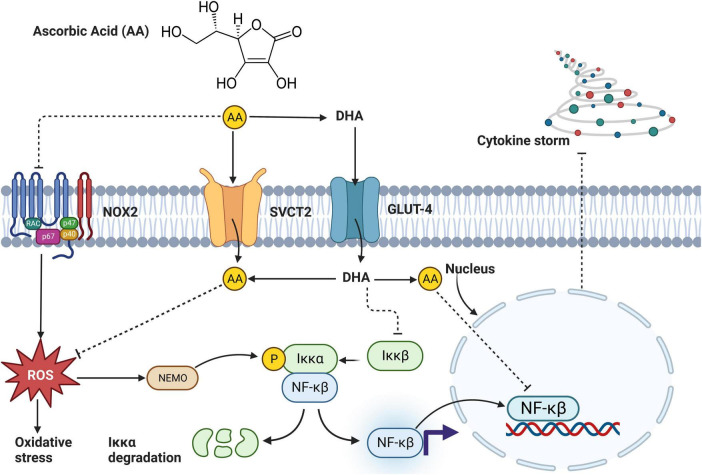
An overview mechanism by which ascorbic acid (AA) found in *Corchorus olitorius* reduces inflammation. AA enters the cell through sodium-vitamin C cotransporter 2 (SVCT2) or indirectly as dehydroascorbic acid (DHA). In the cell, DHA is converted to AA and directly inhibits activation of NF-κβ or through a series of pathways involving suppressing reactive oxygen species (ROS) generation and slowing down oxidative stress. Alternatively, AA blocks NADPH oxidase (NOX2) activation, further inhibiting ROS generation, and oxidative stress. Inhibition of NF-κβ directly inhibits inflammation (102).

With regards to inflammation, a study by Lee et al. (37) showed that 8-week treatment with 4 mg/kg of *C. olitorius* extract in the HFD-induced obese C57BL/6N mouse model significantly (*p* < 0.05) decreases inflammatory markers. These results are further supported by Gomaa et al. (48), who reported a significant decrease in IL-1β (*p* < 0.01) and TNF-α (*p* < 0.01) in obese Sprague–Dawley albino rats treated with *C. olitorius extract* at a dose of 300 or 400 mg/kg for 8 weeks (48). These results support the potential effect of this plant as an anti-inflammatory agent (96), and the effects seem to be mediated by active compounds found in this plant, such as thiamine (97) and riboflavin (98). These compounds reduce inflammation by inhibiting nitric oxide (NO), cyclo-oxygenase (COX), and ROS tyrosine enzymes (99). On the other hand, AA present in *C. olitorius* attenuates inflammation by inhibiting oxidative stress, Iκκ-αβ, NADPH oxidase, NF-κβ, and cytokine storm (Figure 5). Additionally, the ionone and glycosides found in the leaves of *C. olitorius* also show an inhibitory effect on histamine, thus reducing inflammation (100). *C. olitorius* reduces inflammation by inhibiting NO production, and this mechanism is mediated by corchori fatty acids present in this plant (101). The evidence suggests that *C. olitorius* leaves may be an excellent alternative nutraceutical supplement against T2D-related inflammation (Figure 5 and Table 1).

## 10. The safety profile of *C. olitorius* for consumption or treatment

Although the toxicological reports are limited, the available evidence suggests that the *C. olitorius* plant is safe, as no severe adverse events have been reported in rodent models of obesity and T2D. For instance, experiments that investigated the toxicity of *C. olitorius* extract using different doses (50, 100, 200, and 400 mg/kg) in Swiss albino mice showed no adversity in all doses, and there were no changes in the behavioral pattern or any mortality reported (103, 104). Thus, it is evident that the *C. olitorius* treatment is not toxic, as low and high doses were used in the experiments and still showed no evidence of adversity. Our current review showed that no cases of adversities were reported when *C. olitorius* was administered at a low or high dose.

## 11. Overall evidence, concluding remarks, and future directions

Although there are widely used anti-diabetic pharmaceutical drugs, remedies derived from natural antioxidant plants increasingly show positive outcomes when regulating glycemic and lipid metabolism, thus gaining scientific interest. This is attributable to their antioxidant properties and the fact that they are not toxic and have no side effects compared to widely used diabetic drugs. Evidence from pre-clinical experiments in the current review stipulates that *C. olitorius* reduced various pro-inflammatory cytokines, hyperglycemia, and oxidative stress to ameliorate obese and T2D-associated secondary complications. Evidence presented in Table 2 supports the view that *C. olitorius* administration at a low dose of between 2 and 4 mg/kg for 8 weeks in obese could improve inflammation by reducing inflammatory cytokines such as TNF-α, IL-6, and IL-8 concomitant to reducing lipid peroxidation. Similarly, a high dose of 300–400 mg/kg of *C. olitorius* for 8 weeks in obese mice could improve inflammation by significantly reducing plasma IL-1β and TNF-α. Consistent results showed a beneficial effect of *C. olitorius* in regulating glucose metabolism, as demonstrated by a significant reduction in FBG.

Interestingly, *C. olitorius* administration in obese and T2D models improved lipid metabolism, as revealed by a significant decrease in TG, TC, and LDL-c. These results are interesting, considering that many individuals with obesity and T2D develop CVD-related complications, which remain at high risk for mortality worldwide. Therefore, using a natural treatment with anti-hyperglycemic, inflammatory, while reducing dyslipidemia and oxidative stress without side effects, may help reduce the burden of CVD in the obese and T2D population. Although the anti-hyperglycemic effect of this plant was notable in T2D rodents, conflicting results are still observed in the obese model. According to our knowledge, no clinical evidence has been published exploring the effect of this plant on obese and diabetic patients. Hence, it would be essential to examine the antioxidant properties of this plant in these populations, given that the results obtained in pre-clinical studies are promising. While such therapeutic benefits and efficacy are acknowledged in pre-clinical studies, additional evidence is still required to establish the safety profile of *C. olitorius* in rodent models of obesity and diabetes. Moreover, research is still warranted to establish methods to effectively improve its absorption and bioavailability, including establishing the desirable doses and ascertaining its efficacy and safety in obesity and T2D population.

## Author contributions

KM: conceptualization, writing—original draft, data curation, and methodology. KM and WP: writing—review and editing. Both authors approved the final version of the manuscript.
