# Supplementary material for: Corchorus olitorius extract exhibit anti-hyperglycemic and anti-inflammatory properties in rodent models of obesity and diabetes mellitus
Source: Front Nutr. 2023 Apr 5;10:1099880. doi: 10.3389/fnut.2023.1099880 (PMC10113448; doi:10.3389/fnut.2023.1099880)
Supplement: Supplementary file 1 [file Data_Sheet_1.docx]

**Table 1A: Search strategy adapted on PubMed**

| **MeSH no** | **MeSH** | **Hits** |
| --- | --- | --- |
| 1 | Jute leaves | 24 |
| 2 | Corchorus olitorius | 157 |
| 3 | Molokhia | 0 |
| 4 | Obesity | 255610 |
| 1,2,3,4 | (((Jute leaves[MeSH Terms]) OR (Corchorus olitorius)) OR (Molokhia[MeSH Terms])) AND (Obesity[MeSH Terms]) | 4 |

**Table 1B: Search strategy adapted on PubMed**

| **MeSH no** | **MeSH** | **Hits** |
| --- | --- | --- |
| 1 | Jute leaves | 24 |
| 2 | Corchorus olitorius | 157 |
| 3 | Molokhia | 0 |
| 4 | Diabetes | 255610 |
| 1,2,3,4 | (((Jute leaves[MeSH Terms]) OR (Corchorus olitorius)) OR (Molokhia[MeSH Terms])) AND (Diabetes[MeSH Terms]) | 1 |

**Table 1C: Search strategy adapted on Scopus**

| **MeSH no** | **MeSH** | **Hits** |
| --- | --- | --- |
| 1 | Jute leaves |  |
| 2 | Corchorus olitorius |  |
| 3 | Molokhia |  |
| 4 | Obesity |  |
| 1,2,3,4 | (TITLE-ABS-KEY (Jute leaves) OR TITLE-ABS-KEY (Corchorus olitorius) OR TITLE-ABS-KEY ( molokai) AND TITLE-ABS-KEY ( Obesity) ) | 7 |

**Table 1D: Search strategy adapted on Scopus**

| **MeSH no** | **MeSH** | **Hits** |
| --- | --- | --- |
| 1 | Jute leaves |  |
| 2 | Corchorus olitorius |  |
| 3 | Molokhia |  |
| 4 | Diabetes |  |
| 1,2,3,4 | (TITLE-ABS-KEY (jute AND leaves) OR TITLE-ABS-KEY ( corchorus AND olitorius) OR TITLE-ABS-KEY (molokai) AND TITLE-ABS-KEY (diabetes) ) | 21 |

**Table 1E: Search strategy adapted On Semantic Scholar**

| **Terms no1** | **Terms** | **Hits** |
| --- | --- | --- |
| 1 | Corchorus olitorius |  |
| 2 | Obesity |  |
| Combined | “Corchorus olitorius in obesity” | 8 |

**Table 1F: Search strategy adapted on Semantic Scholar**

| **Terms no1** | **Terms** | **Hits** |
| --- | --- | --- |
| 1 | Corchorus olitorius |  |
| 2 | Diabetes |  |
| Combined | “Corchorus olitorius in Diabetes” | 24 |

Additional record through manual screening

(n =4)

Records removed *before screening*: n =0

Duplicate records removed (n = 26)

Records identified from*: (n=113)

PubMed (n = 5)

Scopus (n = 28)

Google Scholar (n = 48)

Semantic Scholar (n = 32)

**Identification**

Records excluded**

Irrelevant title and abstract (n = 30)

Records screened

(n = 87)

**Screening**

Records not retrieved

(n = 0)

Records sought for retrieval

(n =57)

Records excluded: n=41

Phytochemical studies (n = 13)

Human study (n = 1)

Invitro studies (n = 7)

Reviews (n = 12 )

Not on obese or T2D model(n =8)

Records assessed for eligibility

(n = 57)

Studies included in review

(n = 20)

**Included**

Figure 1: PRISMA flow diagram, showing study selection protocol.
